# Network perturbation by recurrent regulatory variants in cancer

**DOI:** 10.1371/journal.pcbi.1005449

**Published:** 2017-03-23

**Authors:** Kiwon Jang, Kwoneel Kim, Ara Cho, Insuk Lee, Jung Kyoon Choi

**Affiliations:** 1 Department of Bio and Brain Engineering, KAIST, Daejeon, Republic of Korea; 2 Department of Biotechnology, College of Life Science and Biotechnology, Yonsei University, Seoul, Republic of Korea; University of Maryland Baltimore County, UNITED STATES

## Abstract

Cancer driving genes have been identified as recurrently affected by variants that alter protein-coding sequences. However, a majority of cancer variants arise in noncoding regions, and some of them are thought to play a critical role through transcriptional perturbation. Here we identified putative transcriptional driver genes based on combinatorial variant recurrence in *cis*-regulatory regions. The identified genes showed high connectivity in the cancer type-specific transcription regulatory network, with high outdegree and many downstream genes, highlighting their causative role during tumorigenesis. In the protein interactome, the identified transcriptional drivers were not as highly connected as coding driver genes but appeared to form a network module centered on the coding drivers. The coding and regulatory variants associated via these interactions between the coding and transcriptional drivers showed exclusive and complementary occurrence patterns across tumor samples. Transcriptional cancer drivers may act through an extensive perturbation of the regulatory network and by altering protein network modules through interactions with coding driver genes.

## Author summary

Identifying driver variants is a current challenge facing cancer genomics. A well-established and robust method for this is to find recurrence in large cohorts of samples. Recurrence patterns of amino acid-changing variants can reveal oncogenes and tumor suppressor genes. However, such single-gene approaches have limitations because of rare variants. Therefore, recurrently affected protein complexes, network modules, or signaling pathways have been identified based on network-level recurrence. Here we dissect chromatin interactome to identify *cis*-regulatory variants that show high gene-level recurrence. We then employ the gene regulatory network and protein interactome to characterize putative cancer genes with *cis*-regulatory variant recurrence. These genes were located at critical positions in the regulatory network. By contrast, they are at the circumference in the protein interactome; instead, they form a network module with coding cancer genes located at hub positions. Furthermore, the coding and regulatory variants associated via these interactions showed exclusive and complementary occurrence patterns across tumor samples. Therefore, we suggest that transcriptional cancer drivers may act through an extensive perturbation of the regulatory network and by altering protein network modules through interactions with coding driver genes.

## Introduction

Recent efforts to understand noncoding variation through epigenomic annotation have shown that disease-associated variation is frequently located in regulatory DNA marked by DNase I hypersensitive sites (DHSs) or particular histone modifications [1–4]. Noncoding somatic variants in cancer have been a focus of interest since the recent discovery of TERT promoter variants [5,6], which was followed by efforts to systematically analyze the whole noncoding genome [7–9]. Epigenomic dissection of cancer genomes revealed that chromatin accessibility and histone modifications in corresponding cell types shape the noncoding variant landscape [10]. DNA repair activity was found to be a determinant of variant density within DHSs [11–13].

Identifying driver variants is one of the greatest challenges currently facing cancer genomics. Probably the most robust way to find driver variants is by leveraging large cohorts of samples and using recurrence as an indicator of selection [14]. Efforts to identify recurrent variants in cancer have focused on protein-coding sequences. However, a sizeable fraction of tumor samples lack variants in highly recurrent genes, indicating that the single gene-based approach may miss a large number of true driver genes [14]. In this light, protein interaction networks or signaling pathways were dissected to identify drive modules or driver pathways based on a combinatorial recurrence of coding variants [15–19].

Inferring the driver status of noncoding variants can be more complicated than coding variants. Noncoding recurrence was previously examined within single promoters or at the same sites. However, a majority of variants reside in distal enhancers, which scatter across a long distance while converging on the same target transcript. Therefore, target gene identification is crucial for estimating regulatory variant recurrence. To this end, it is essential to determine three-dimensional chromatin structure [20]. For example, a novel metabolic regulator was discovered by surveying long-range interactions that engage an obesity-associated variant [21].

From breast and liver cancer genomes, we first identify regulatory driver variants and their associated genes, referred to as transcriptional drivers (TDs), based on combinatorial recurrence over the chromatin interactome. We then characterize the TD genes at the systems level in comparison with coding driver (CD) genes by projecting them onto the gene regulatory network and protein interactome. In particular, we utilize a Bayesian network that models causal (directional) regulatory relationships [22], a transcription network that contains direct co-regulatory interactions [23], an integrated physical protein interaction network [24], and a probabilistic functional protein association network [25].

## Results/Discussion

**Fig 1:**
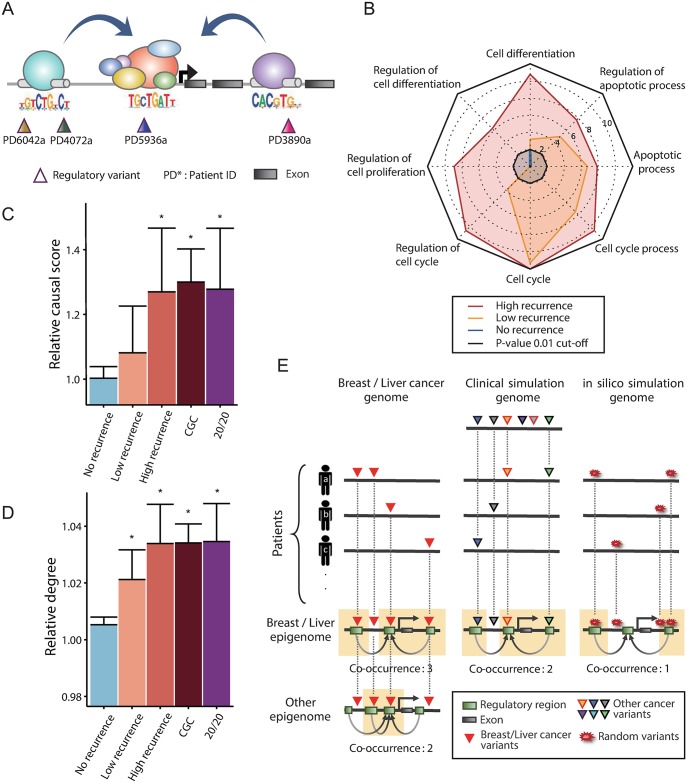
Combinatorial *cis*-regulatory recurrence. (A) Illustration of our recurrence model. Four variants from different samples are scattered in *cis*-regulatory regions but converge on the same gene via chromatin interactions. (B) A radar plot showing the significance of enrichment for eight cancer-related Gene Ontology terms. The length of the plot scales with log_10_ (P value). The P values were derived from the hypergeometric distribution and adjusted for multiple testing by the Bonferroni correction. (C) Relative causal score of the TDs grouped by the recurrence level and the CDs (CGC and 20/20) in the Bayesian network of breast cancer. Causal scores were calculated as described in the Methods and normalized by dividing by the average causal score of all genes in the network. (D) The relative degree of the TDs and CDs in the coexpression network in breast cancer. The degree was divided by the network average. (E) Schematic illustration of genomic simulation (*in silico* or clinical) in which variants are randomized, and epigenomic simulation in which K562 chromatin interactome is used in place of MCF-7 and HepG2.

The workflow of our analyses is summarized in S1 Fig. We first identified regulatory variants in 119 breast and 88 liver cancer samples as illustrated in Fig 1A. In this example, four different samples carry motif-changing variants at different positions in cis-regulatory regions, whose convergence on a common transcriptional target is revealed by the chromatin interactome. In this case, the combinatorial measure of variant recurrence for this gene should be four although none of the four variants arose at the same site. For this type of recurrence analysis, we employed enhancer-promoter maps constructed by RNA polymerase II-mediated chromatin interaction analysis by paired-end tag (ChIA-PET) sequencing [26–28], integrated methods for predicting enhancer targets (IM-PET) [29], DHS tag density correlations [1], and cap analysis gene expression (CAGE)-based RNA correlations [30]. We also applied additional filters for enhancer-promoter mapping (see Methods). The different criteria and resulting number of chromatin interactions are described in S2 Fig. The list of genes with the resulting recurrence level in each cancer is provided in S1 Table.

The genes recurrently mutated in this manner (i.e., TD genes) in either breast or liver cancer were enriched for cancer-related biological processes such as cell cycle, differentiation, and apoptosis (Fig 1B). This enrichment was more pronounced with higher recurrence than lower recurrence. In addition, these genes appeared to play a highly causative function in the cancer regulatory network. When applied to the directional regulatory network in breast cancer [22], the TDs of breast cancer exhibited a high causal score; in other words, they have a relatively high outdegree in the network while positioned upstream of the causal path (Fig 1C; see Methods). This means that they tend to exert regulatory effects rather than be regulated by other genes. Their causal score was so high as the known CDs that were identified based on the 20/20 rule [14] or retrieved from the Cancer Gene Census (CGC) database [31]. This pattern was not found when the TDs of liver cancer were projected onto the breast network (S3A Fig). We also constructed other types of regulatory networks that show regulatory associations but not regulatory directions [23,32,33] for each cancer. Again, a high connectivity of the TDs was observed (Fig 1D, S3B~S3D Fig). Taken together, the TDs identified based on combinatorial cis-regulatory variant recurrence seem to play a crucial oncogenic role through an extensive perturbation of the regulatory network.

We performed permutation-based statistical tests on the cis-recurrence measures (Fig 1E). First, two types of variant simulations were performed. We first randomly generated the same number of variants while maintaining the distribution of per-sample variant counts (in silico simulation). We also performed a clinical simulation in which the same number of variants was retrieved from the control set of variants derived from samples of other cancer types. In both breast cancer and liver cancer, and from both simulations, the observed level of recurrence was significantly higher than that expected by chance (S4 Fig). Selected examples of individual genes are provided in S5 Fig. Next, we mapped variants to an irrelevant chromatin interactome with comparable data types and size (“Other epigenome” in Fig 1E). Based on K562 data, we generated control epigenomic datasets against MCF7 and HepG2. In contrast to non-recurrent genes, recurrently mutated genes were 2~3 times more frequently detected when using the matched epigenome (S6 Fig), implying the tissue specificity of the recurrent cis-regulatory variants. Together, these results suggest the combinatorial recurrence patterns we identified were of biological relevance rather than from technical artifacts.

**Fig 2:**
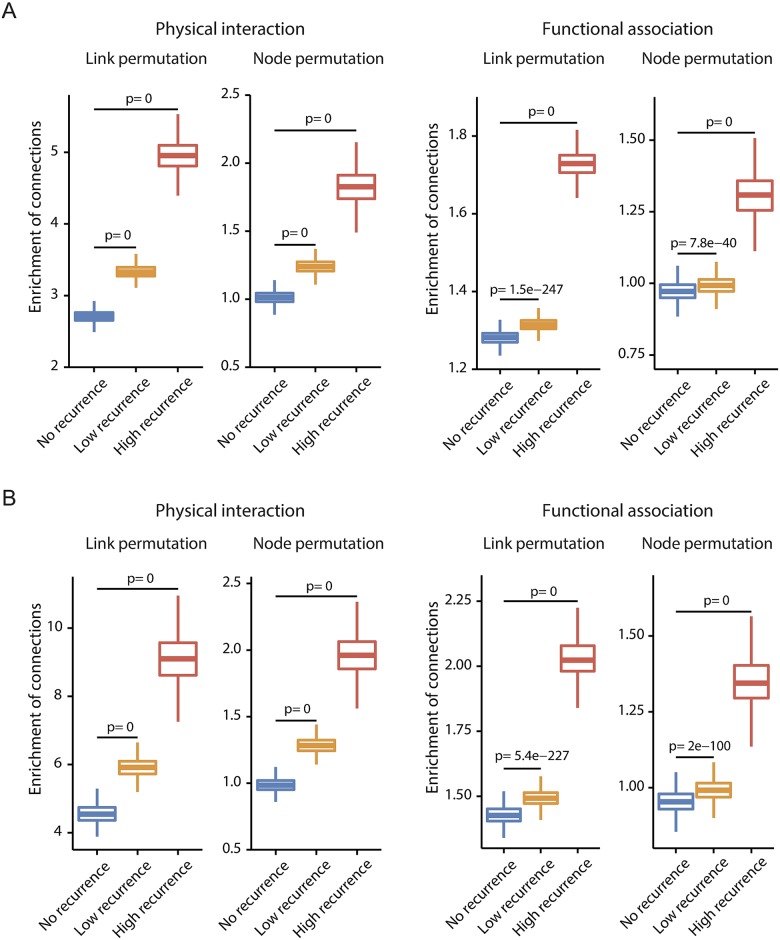
Overrepresentation of interactions between coding drivers and transcriptional drivers in the protein interactome. The significance of enrichment was estimated as the observed-to-expected ratio of the number of interactions for each TD category grouped by the recurrence level as combined for breast and liver cancer. The expected number was obtained by permuting the links or nodes of the network. The permutation was repeated 1,000 times. (A) Enrichment of interactions between the TDs and CGC CDs. (B) Enrichment of interactions between the TDs and 20/20 CDs.

The 20/20 and CGC CDs also showed high connectivity in the transcription network despite some exceptions (S3B Fig), meaning that they may be able to act through transcriptional perturbation as well as through protein malfunction. By contrast, in the protein-protein interaction network [24] and functional association network [25], the TDs were not so highly connected as the CDs (S7 Fig). These suggest that unlike the CDs, the oncogenic effects of the TDs may be confined to the transcription network. However, disease proteins are not scattered randomly in biological networks, but tend to interact with each other and form network modules [34]. Therefore, we tested whether the TDs frequently interact with the CDs in the protein interactome. Indeed, we observed a positive correlation between the recurrence level of the TDs and their agglomeration with the CD genes (Fig 2 and S8 Fig). In other words, genes with a high recurrence of regulatory variants tend to interact frequently with genes with a high recurrence of coding variants.

**Fig 3:**
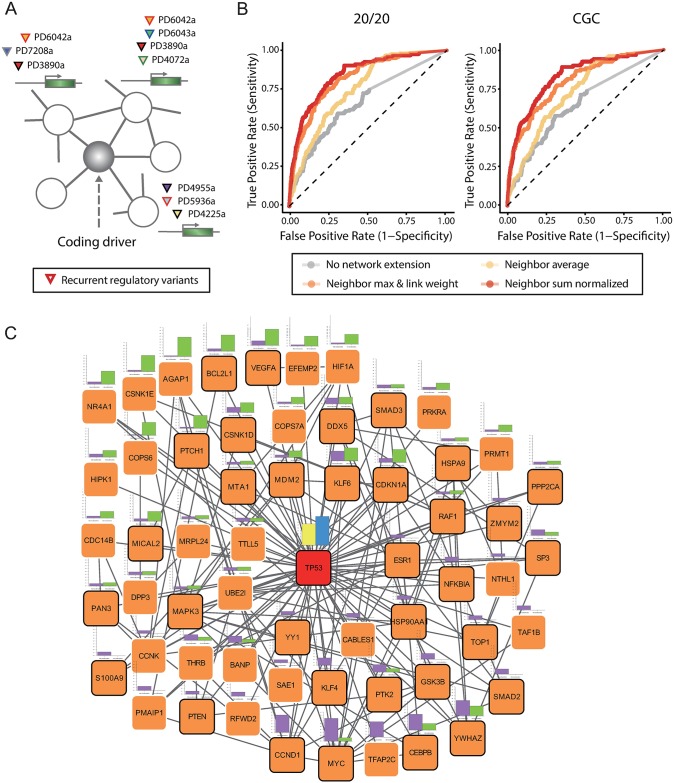
Network module of coding drivers and transcriptional drivers. (A) Schematic view of a network module consisting of the central CD and its partner TDs. (B) ROC graphs for the prediction of the 20/20 CD (left) and CGC CD (right) based on the modular recurrence level. The gray curves are results when the *cis*-regulatory recurrence level of the CD alone was used. The colored curves are resulted from a modular extension of recurrence based on the average, sum, or maximum of the neighbor TDs (see Methods for detail). (C) Network-level recurrence patterns of the TP53 module. The yellow and blue bars at the center indicate the coding recurrence levels of TP53 in breast cancer and liver cancer, respectively. The violet and green bars at the circumferences represent the regulatory recurrence levels of TP53-interacting genes in the functional network in breast cancer and liver cancer, respectively.

This finding, in concert with the high degree of the CDs in the protein interactome, led us to test whether the CDs have modular relationships with the TDs (Fig 3A). For a given gene and all its neighbors in the network, we computed the combinatorial chromatin-based measure of cis-recurrence as described above. Then, we examined the degree to which the cis-recurrence levels of the given gene itself and all its neighbors can predict the coding driver status of the given gene (see Methods). The CDs themselves had a higher level of cis-recurrence than other genes as indicated by the gray receiver operating characteristic (ROC) curves in Fig 3B. This is consistent with the high connectivity of the CDs in the regulatory network. However, the modular extension of the recurrence levels considerably improved the performance of CD prediction (colored ROC curves in Fig 3B). The TP53 network module is illustrated in Fig 3C with the coding recurrence levels in breast and liver cancer (yellow and blue bars at the center) and regulatory recurrence levels in each cancer (violet and green bars at the circumferences).

**Fig 4:**
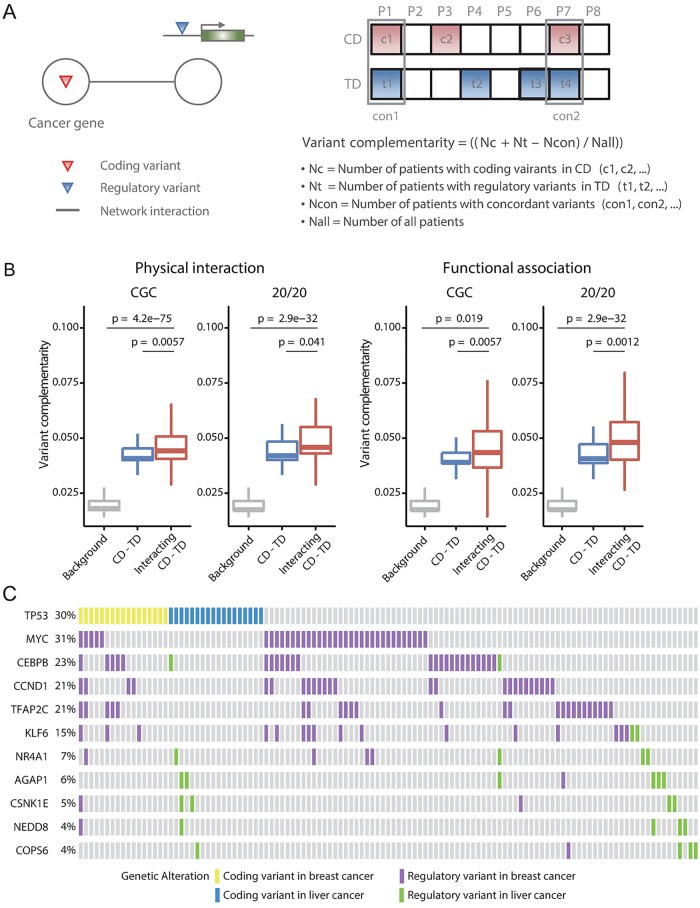
Complementary recurrence of variants of coding drivers and interacting transcriptional drivers. (A) Schematic illustration and description of the variant complementarity measure between the interacting CD and TD. (B) Variant complementarity of interacting CD-TD pairs (red boxplots), all CD-TD pairs (blue boxplots), and all pairs (inclusive of non-recurrent genes) with background coding-regulatory variants (gray boxplots). (C) Complementary recurrence patterns between coding variants of TP53 and regulatory variants of top 5 genes with highest complementarity in breast cancer and top 5 genes with highest complementarity in liver cancer. Each column indicates each breast or liver cancer sample. Regarding variant types, the same color-coding as in Fig 3C was used.

It is notable that this approach performs better for the prediction of the CDs than for the prediction of all known cancer genes (S9 Fig). For example, compare the CGC CDs identified by point variants (Fig 3B) with all CGC genes (S9 Fig). This implies evolutionary interactions between protein-coding and cis-regulatory point variants during cancer development. We examined complementary recurrence patterns of interacting coding and regulatory variants. We computed variant complementarity as described in Fig 4A for each pair of genes. As shown in Fig 4B, this measure was significantly higher for the interacting CD-TD pairs (red boxplots) than all CD-TD pairs (blue boxplots) and all background coding-regulatory variant pairs (gray boxplots). Complementary variant patterns between coding variants of TP53 and regulatory variants of its interacting genes with the greatest degrees of variant complementarity are illustrated in Fig 4C. In the given breast cancer samples, MYC, CEBPB, CCND1, and TFAP2C regulatory variants showed clear mutual exclusivity between themselves as well as with TP53 coding variants. Mutual exclusivity of the coding variants of proteins on the same signaling pathways has been a focus of interest. However, such relationships between coding and regulatory variants have never been investigated before.

In summary, we search the chromatin interactome and protein interactome for combinatorial regulatory variant recurrence with aim to prioritize cancer-driving genes. Candidate transcriptional driver genes, ones that are recurrently affected by cis-regulatory variants via chromatin interactions, showed functional and network features that could be shared with cancer-driving genes. The gene transcription network, especially the Bayesian causal regulatory network, exhibited the potential effects of these genes on extensive network perturbation. Genes with recurrent coding variants also stood out in the regulatory network. For example, tumor suppressors and oncogenes can perturb the regulatory network through transcriptional silencing or activation. In fact, these genes were high in cis-regulatory recurrence, indicating that they may be recurrent for both coding and regulatory variants.

For the first time, we systematically investigated interactions between genes associated with coding variants and those with regulatory variants. The regulatory recurrent genes are not hubs per se in the protein interactome but frequently interact with genes of high coding variant recurrence. The variant occurrence patterns support the complementary evolution of the coding and interacting regulatory variants during cancer development. Therefore, the recurrent regulatory variants may act not only through an extensive perturbation of the regulatory network but also by altering the protein network through interactions with coding variants.

To directly estimate the effect of a *cis*-regulatory variant, the regulatory network of the sample that carries the given variant should be interrogated. For example, a personalized characterization of regulatory variants can be conducted by using sample-specific networks [35]. This approach will be useful when one is interested in a specific driver gene and would like to know which particular genes are affected by the variants of this driver gene. For this, we need a large number of whole-genome sequenced samples from which TDs can be identified reliably and matched gene expression data based on which sample-specific networks can be constructed.

It should be noted that recurrence is not an absolute indicator of cancer-driving variants. For example, harmfulness of amino acid substitutions can be directly measured [36]. Cancer-related genes identified in this fashion showed high connectivity in protein interaction networks [36] as the CDs identified on the basis of recurrence. However, there is currently no such method for noncoding regulatory variants. In conclusion, our results illustrate that various types of biological networks can deepen our understanding of the cancer genome and promote the discovery of novel cancer genes.

## Material and methods

### Sequencing data acquisition and processing

We downloaded variant calls for whole genome sequences of 507 cancer samples across 10 different cancer types from ftp://ftp.sanger.ac.uk/pub/cancer/AlexandrovEtAl/somatic_mutation_data [37]. The variants detected by the filters of the Sanger pipeline were excluded from our analysis. In total, we used 647,695 point variants in 119 breast cancer samples and 899,449 point variants in 88 liver cancer samples. Variants of other tumor types were used for our clinical simulation, in which the same number of variants as in breast or liver cancer were retrieved and subjected to the computation of combinatorial recurrence. To single out functional variants, we applied the position weight matrix of transcription factor binding to the variant sites. The transcription factor binding information was obtained from the human CIS-BP (Catalog of Inferred Sequence Binding Preferences) database [38] and TRANSFAC [39]. The screening of transcription factor binding sites was performed by the FIMO tool [40]. Gain or loss of the binding sites by variants was evaluated based on the P value differences from the FIMO outputs. The P value cutoff of 10–5 was used.

### Chromatin interactome data

To map the target genes of the identified regulatory variants, we used four chromatin interactome datasets in each cancer type: (1) ChIA-PET [26–28], (2) Distal-proximal DHS tag correlation [1], (3) CAGE-based enhancer RNA-messenger RNA correlation [30] and (4) IM-PET [29]. As for ChIA-PET, we focused on RNA polymerase II-mediated chromatin interactions measured in MCF-7 and K562 [27]. For a filtering purpose, PET counts ≥ 3 were used to avoid false positive interactions. CAGE-detected enhancer RNA varied 2 ~ 2,860 bp in length, so we defined enhancers as 100 bp upstream and downstream of the center of an enhancer RNA. For gene annotation, we used protein coding genes from the GENCODE v19 [41]. Promoters were defined as 2 kb upstream ~ 500 bp downstream of the transcription start site. Finally, we merged all chromatin interactome data separately for each cell type (MCF-7, HepG2, and K562) after filtering out promoter-promoter interactions. Because the DHS correlation and CAGE correlation data provide a universal set of enhancer-promoter mappings, we intersected the DHS regions of the relevant cell type to reconstruct the cell type-specific chromatin interactome. The cell type-specific subsets were combined with ChIA-PET and IM-PET in MCF7 for breast cancer analysis, IM-PET in HepG2 for liver cancer analysis, and ChIA-PET and IM-PET in K562 for the epigenome simulation described later.

### Identifying recurrent regulatory variants

We computed combinatorial cis-regulatory recurrence levels by projecting the regulatory variants in breast cancer and liver cancer onto the merged chromatin interactome. Recurrent genes were defined as having a co-occurrence of > = 2. Furthremore, we grouped all genes into three categories according to their combinatorial cis-recurrence levels: no recurrence for a co-occurrence of 1, low recurrence for a co-occurrence of 2 ~ 4, and high recurrence for a co-occurrence of > = 5. The two regions linked by chromatin interactome data per se can be assumed to be cis-regulatory regions; the ChIA-PET, DHS correlation, CAGE correlation, and IM-PET interactome are based on RNA polymerase II binding, chromatin accessibility, enhancer and messenger RNA expression, and various enhancer and promoter features, respectively. However, we applied additional filters for the detection of enhancers and promoters by using histone modification (H3K27ac and H3K4me3), RNA polymerase II binding, p300 binding, and RNA expression. The different criteria and resulting number of chromatin interactions are shown in S2 Fig. The resulting recurrence level with each criterion for each gene is provided in S1 Table.

### Recurrence simulation tests

We performed two types of genomic simulation and one type of epigenomic simulation to assess the statistical significance of the observed combinatorial cis-recurrence levels. First, random variant sets were constructed for each cancer in silico by generating the same number of variants while maintaining the distribution of per-sample variant counts. Second, instead of randomly generating in silico variants, the same number of variants for each sample was retrieved from the other 506 clinical samples across various tumor types. These two genomic simulations were repeated 1,000 times to generate a null distribution of recurrence levels. Third, we mapped the real variants to an irrelevant chromatin interactome. The same number of the ChIA-PET, IM-PET, DHS correlation, CAGE correlation interactions as the original data (MCF-7 and HepG2) was retrieved randomly from the matching K562 data.

### Gold-standard sets of coding drivers and other cancer genes

We generated two gold-standard sets of the CDs: one from the CGC database [31] and the other based on the 20/20 rule [14]. From the CGC, we retained frameshift, missense, nonsense, and splicing variants while excluding amplifications, large deletions, and translocations, in order to focus our analysis on point variants. The 20/20 set was constructed based on the hypothesis that > 20% variants in an oncogene should be at recurrent positions and > 20% variants in a tumor suppressor gene need to be inactivating or truncating. We used three more inclusive gene sets. First one was CGCAll, which included all genes in the CGC database. Second, AllOnco is a master set of other seven cancer gene sets [42]. Third, MouseIns is a set consisting of genes identified by insertional mutagenesis in mice [43,44].

### Regulatory network analysis

For a directional, causal gene regulatory network, we employed our previously constructed breast cancer Bayesian network [22]. This global network was constructed at an unprecedented level of biological coverage and accuracy based on precise modeling of genomic regulatory interactions. We computed a causal score for each gene on the basis of its outdegree (the number of outgoing links) in the network and relative position in the causal chain. The causal chain was defined as the longest (or shortest) path connecting the head and tail nodes via the gene of interest. The causal score is proportional to the relative outdegree in the network (the number of outgoing links divided by the total number of links of the given node) and the relative distance to the tail node of the causal path. The relative distance was obtained by considering the length of the causal path. Choice of the longest or shortest path did not make significant differences. For non-directional association networks, we employed ARACNe (Algorithm for the Reconstruction of Accurate Cellular Networks) [23] and PCA-PMI (Part Mutual Information-based PC-algorithm) [33]. We applied the available tools (http://califano.c2b2.columbia.edu/aracne [45] and http://www.sysbio.ac.cn/cb/chenlab/software/PCA-PMI/) for gene expression data in breast cancer and liver cancer separately. For this analysis, we downloaded gene expression data (Illumina HiSeq-based) for 1215 breast cancer and 423 liver cancer samples from the Cancer Genomics Browser (https://genome-cancer.ucsc.edu/).

### Protein interactome analysis

We employed two different types of the protein interactome: (1) an integrated physical interaction network constructed by adding interactions from Stitch-seq mapping [46] and the HINT database [47] to the basal data [24] consisting of the yeast-two-hybrid interaction pairs and integrated literature-based protein-protein interactions, and (2) a probabilistic functional network [25] constructed by a modified Bayesian integration of various types of data from multiple organisms. Only direct links between the CDs and TDs were considered. The expected number of interactions was estimated by generating the null distribution through 1,000 random permutations of nodes or links of each network. The real (observed) number of interactions was divided by the 1,000 randomized (expected) numbers of interactions; thus we calculated enrichment scores as the observed-to-expected ratios.

### Driver prediction by modular regulatory recurrence

We sought to examine modular relationships between the CDs and TDs. First, for a given gene and all its neighbors in the network, we computed the combinatorial chromatin-based measure of cis-regulatory recurrence. Then, we examined the degree to which the cis-recurrence levels of the given gene itself and its neighbors can predict the coding driver status of the given gene. We used three metrics for recurrence combination at the modular level. Let *M*(*v*) be the number of cases (patients) in which gene (node) *v* has *cis*-regulatory variants, in other words, the combinatorial cis-regulatory recurrence of gene *v*. Let *L*(*v*) be the set of linked neighbors of gene *v* and deg(*v*) be the number of linked neighbors of gene *v*. With these, the three scoring metrics for gene *v* are defined as follows.

Average of the neighbor variant occurrences:

$$Score_{average}\left(v\right)=M\left(v\right)+\;\frac{\sum_{u\in L\left(v\right)}M\left(u\right)}{\text{deg}\left(v\right)}.$$

Weighted max of the neighbor variant occurrences:

$$Score_{Max}\left(v\right)=M\left(v\right)+\text{max}\left\{x|x=M\left(u\right)\times W\left(v,u\right),\;u\in L\left(v\right)\right\},$$

where *W*(*v*, *u*) is the normalized edge weights between gene *v* and gene *u*, which indicate the degree of functional association [25].

Degree-normalized sum of the neighbor variant occurrences:

$$Score_{sum}\left(v\right)=M\left(v\right)+\;\sum_{u\in L\left(v\right)}\frac{M\left(u\right)}{\text{deg}\left(u\right)}.$$

Considering the edge weights did not improve the predictability of the method in the case of the average and sum of the neighbor variant occurrences.

## Supporting information

S1 Fig Workflow of the study. (TIF)

S2 Fig (A) Schematic of different filters and (B) resulting number of chromatin interactions. The applied filters were based on histone modification (H3K27ac and H3K4me3), RNA polymerase II binding, p300 binding, and RNA expression.
(TIF)

S3 Fig Characterization of the TDs in the transcription network. (A) Relative causal score of the liver TDs grouped by the recurrence level in the breast cancer network. (B) Relative degree of the liver cancer TDs in the liver cancer coexpression network based on ARACNe. (C) Relative degree of the breast cancer TDs in the breast cancer association network based on PCA-PMI. (D) Relative degree of the liver cancer TDs in the liver cancer association network based on PCA-PMI.
(TIF)

S4 Fig Results of the in silico and clinical simulation for (A) breast cancer and (B) liver cancer variants. The null distribution of the number of recurrently mutated genes (upper) and the average recurrence of all genes (lower) generated by 1,000 simulations. The red and green lines denote the real figures.
(TIF)

S5 Fig Examples of the TDs in (A) breast cancer and (B) liver cancer. The red and green lines indicate the real recurrence level of each gene. The null distribution of the recurrence levels was generated by the in silico or clinical simulation.
(TIF)

S6 Fig Results of the epigenome simulation in (A) breast cancer and (B) liver cancer. The number of non-recurrent (left) and recurrent (right) genes when the matched epigenome (MCF-7 or HepG2) or control epigenome (K562) was used.
(TIF)

S7 Fig Relative degree of the TDs and CDs in (A) the physical interaction network and (B) the functional association network. The breast and liver cancer TDs were combined.
(TIF)

S8 Fig Frequent protein interactions between the TDs and (A) CGC CDs and (B) 20/20 CDs. Link or node randomization was performed 1,000 times to obtain the distribution of expected number of interactions. The red lines denote the observed number of interactions.
(TIF)

S9 Fig ROC graphs for the prediction of non-CD cancer genes based on the modular recurrence level. (TIF)

S1 Table Combinatorial cis-regulatory recurrence levels measured for each gene by using the different criteria described in S2 Fig. (XLSX)

S2 Table The measurement matrix of combinatorial recurrence. (XLSX)
