# Supplementary figures and images for: Network perturbation by recurrent regulatory variants in cancer
Source: PLoS Comput Biol. 2017 Mar 23;13(3):e1005449. doi: 10.1371/journal.pcbi.1005449 (PMC5383347; doi:10.1371/journal.pcbi.1005449)

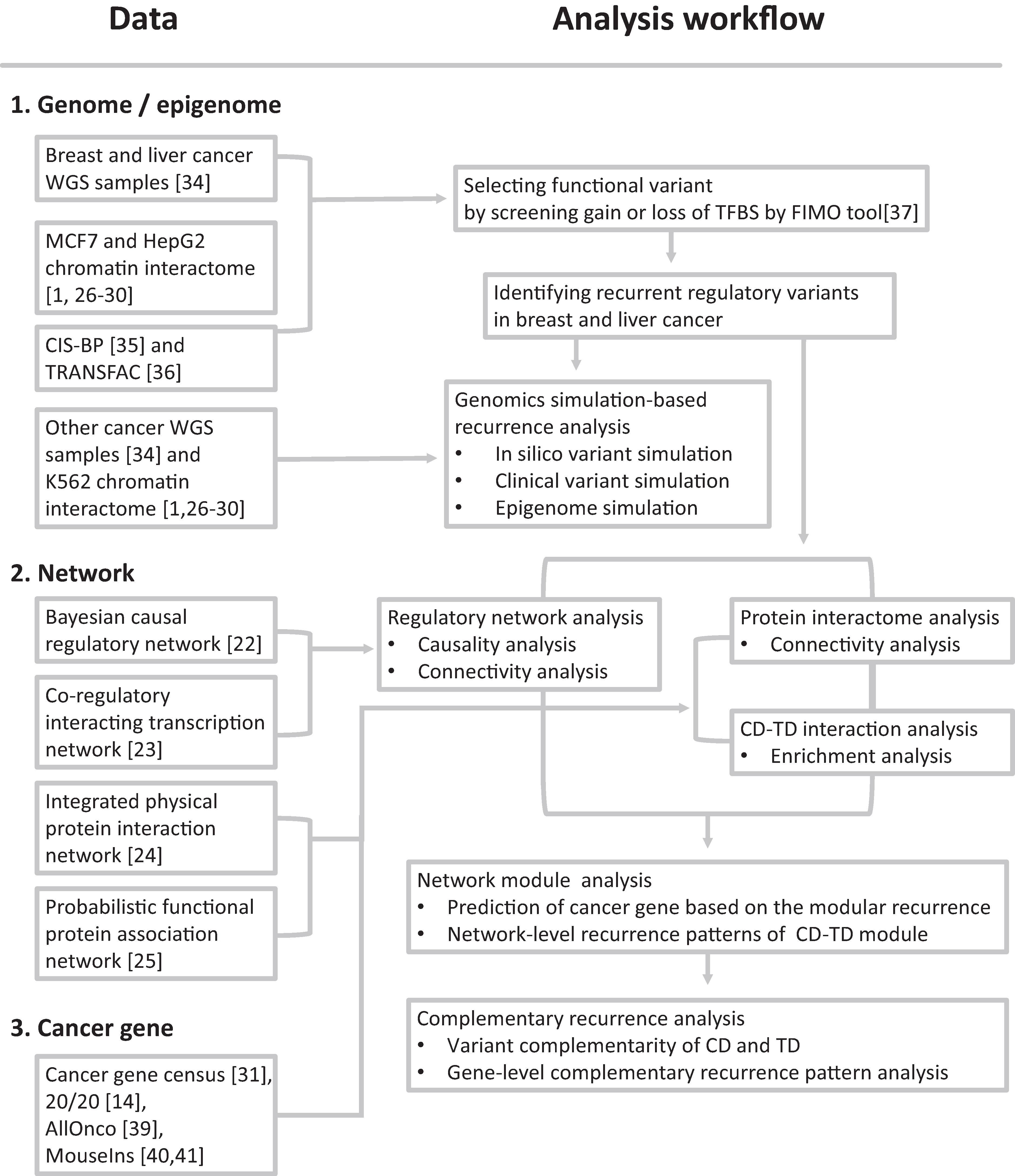

Supplement: S1 Fig — (TIF) [file pcbi.1005449.s001.tif]

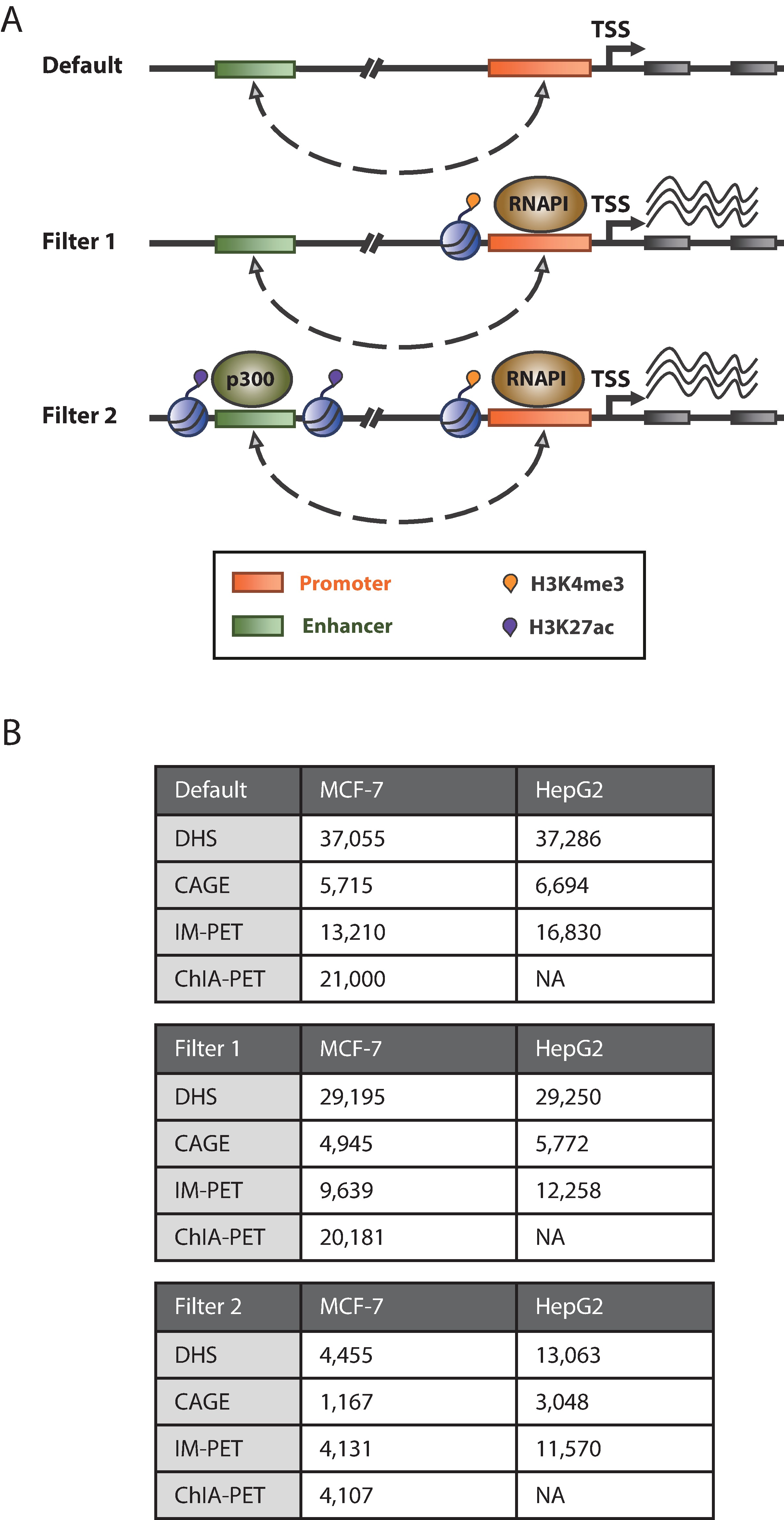

Supplement: S2 Fig — The applied filters were based on histone modification (H3K27ac and H3K4me3), RNA polymerase II binding, p300 binding, and RNA expression. (TIF) [file pcbi.1005449.s002.tif]

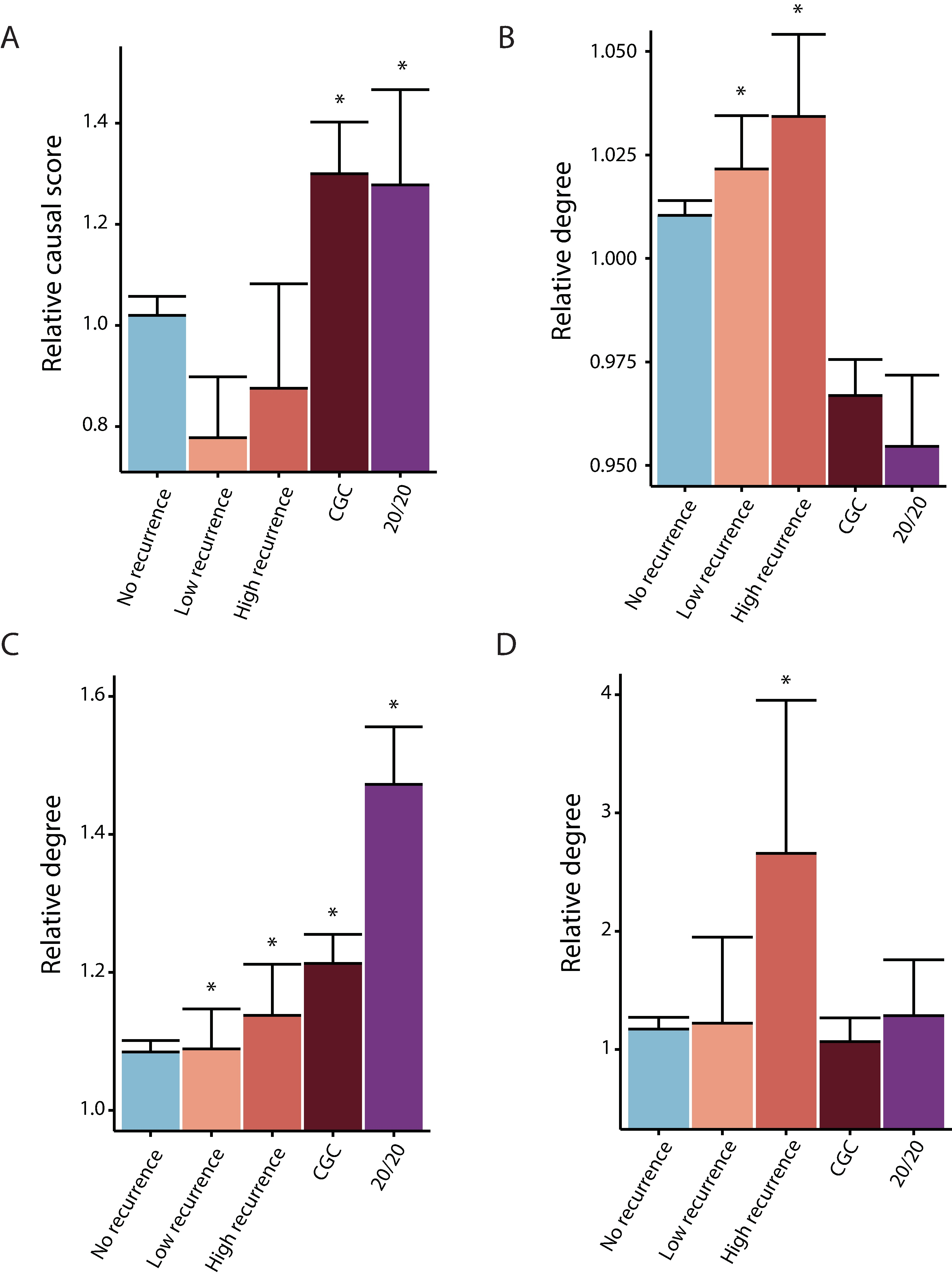

Supplement: S3 Fig — (A) Relative causal score of the liver TDs grouped by the recurrence level in the breast cancer network. (B) Relative degree of the liver cancer TDs in the liver cancer coexpression network based on ARACNe. (C) Relative degree of the breast cancer TDs in the breast cancer association network based on PCA-PMI. (D) Relative degree of the liver cancer TDs in the liver cancer association network based on PCA-PMI. (TIF) [file pcbi.1005449.s003.tif]

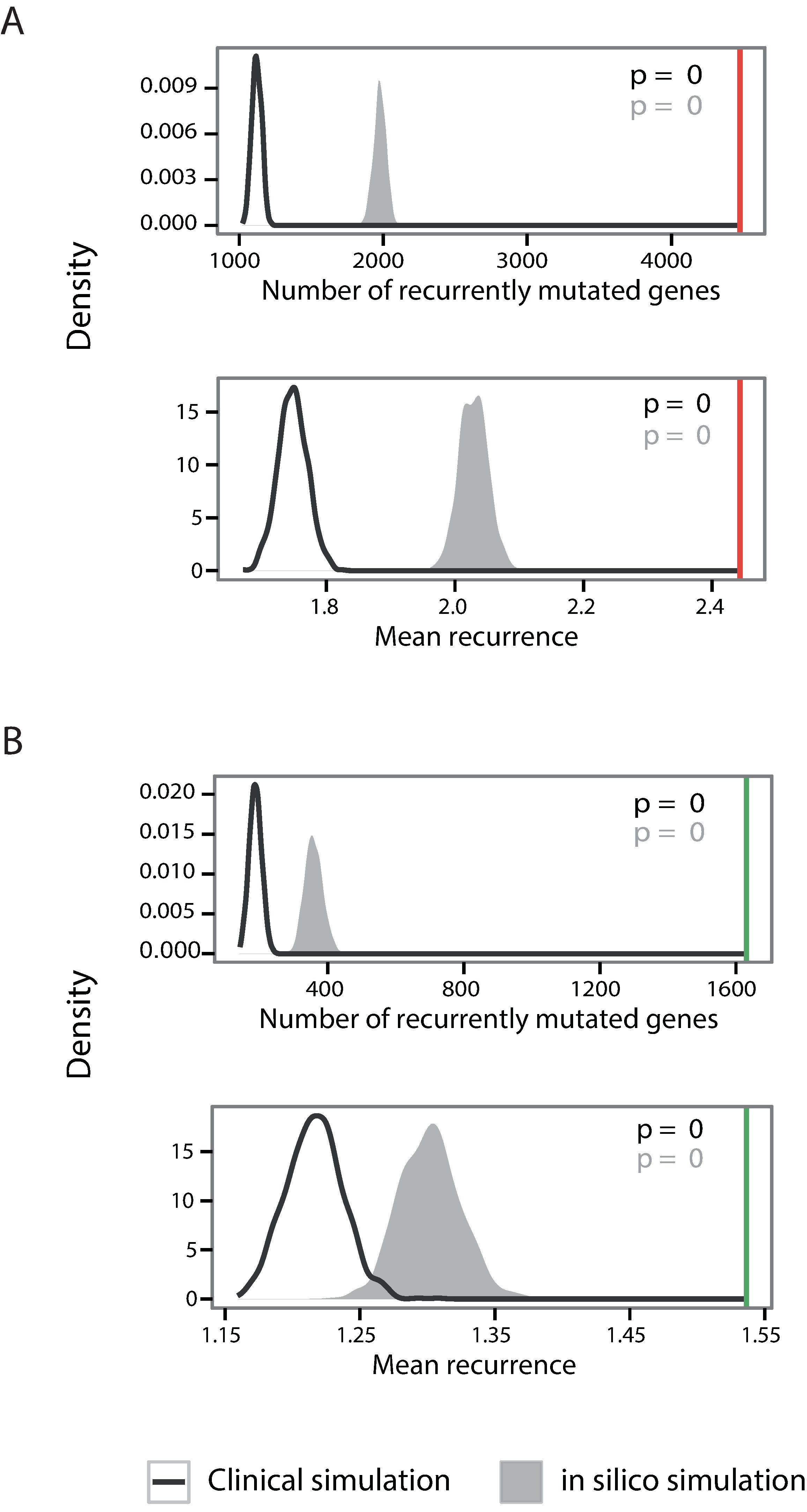

Supplement: S4 Fig — The null distribution of the number of recurrently mutated genes (upper) and the average recurrence of all genes (lower) generated by 1,000 simulations. The red and green lines denote the real figures. (TIF) [file pcbi.1005449.s004.tif]

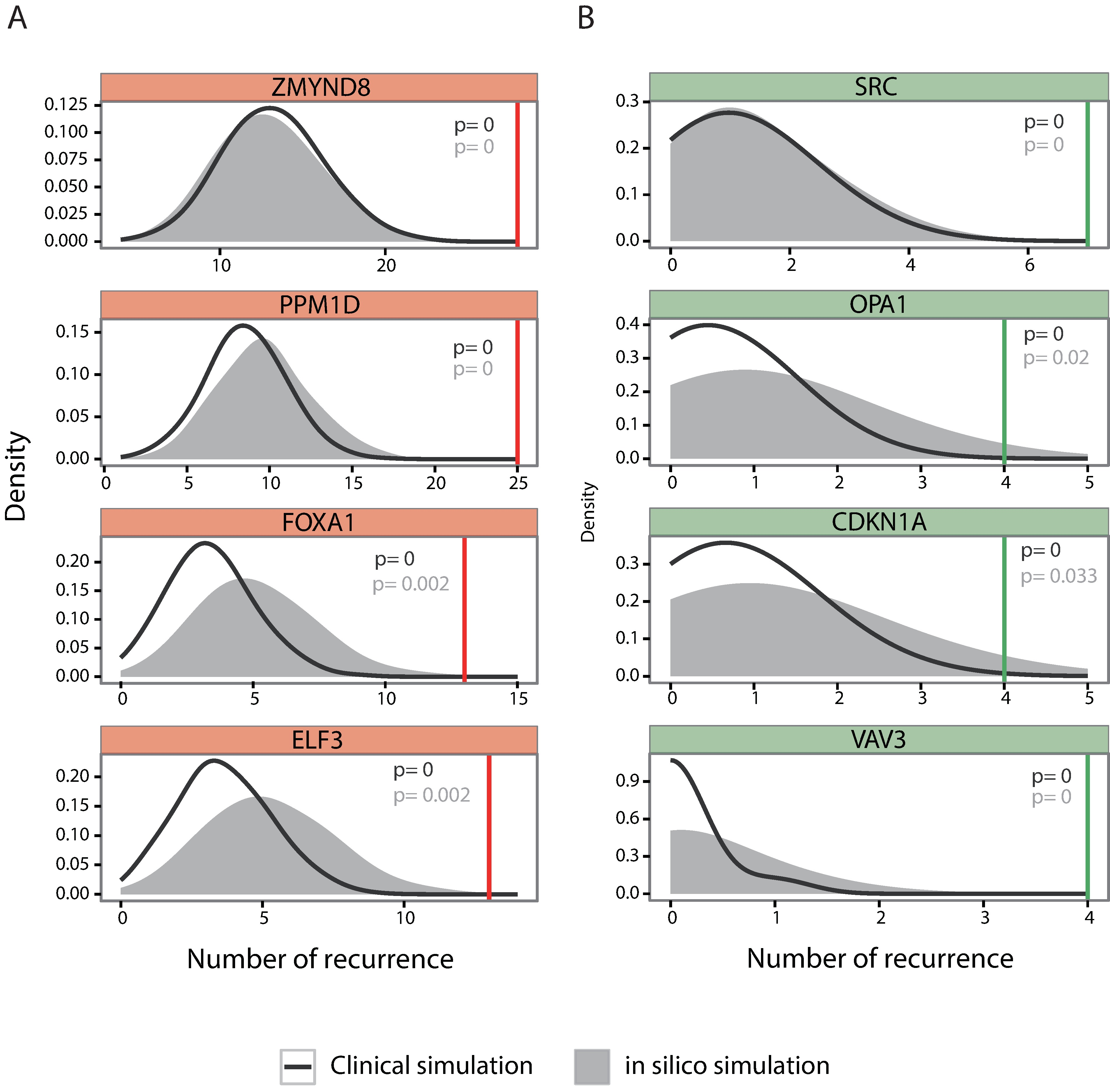

Supplement: S5 Fig — The red and green lines indicate the real recurrence level of each gene. The null distribution of the recurrence levels was generated by the in silico or clinical simulation. (TIF) [file pcbi.1005449.s005.tif]

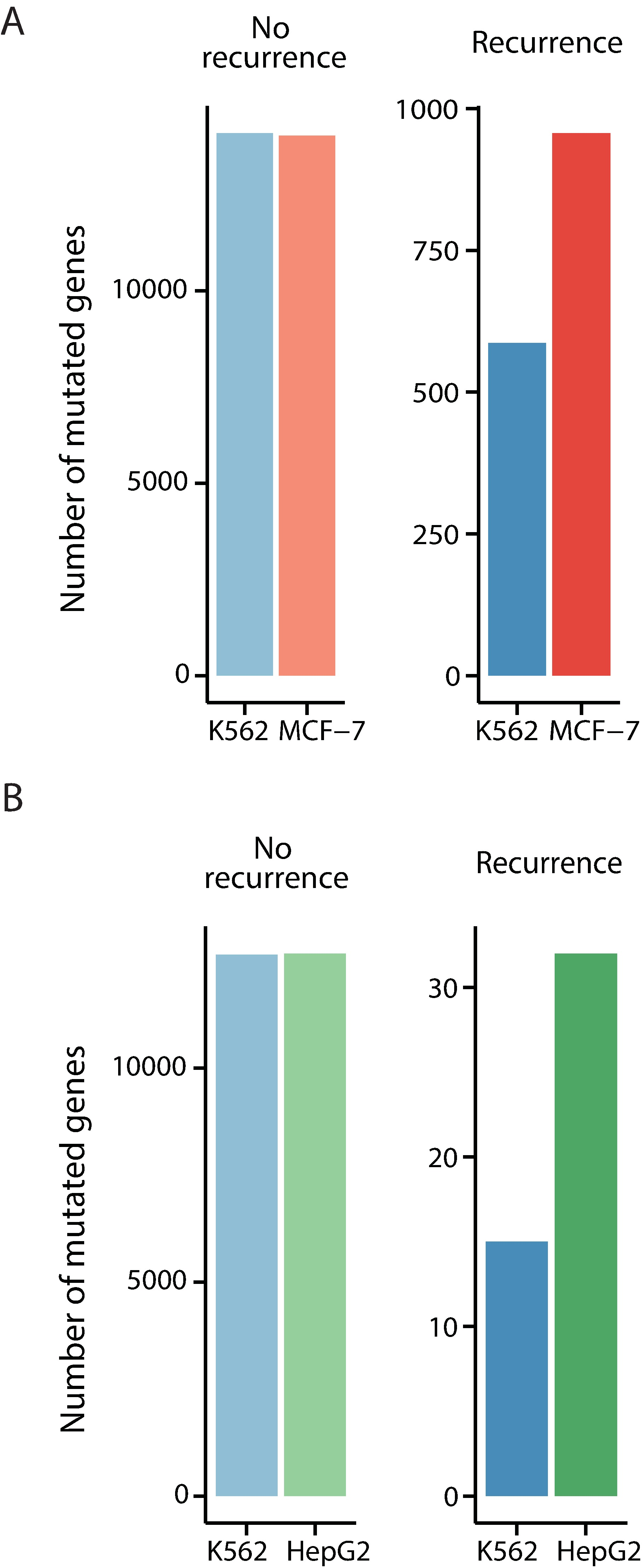

Supplement: S6 Fig — The number of non-recurrent (left) and recurrent (right) genes when the matched epigenome (MCF-7 or HepG2) or control epigenome (K562) was used. (TIF) [file pcbi.1005449.s006.tif]

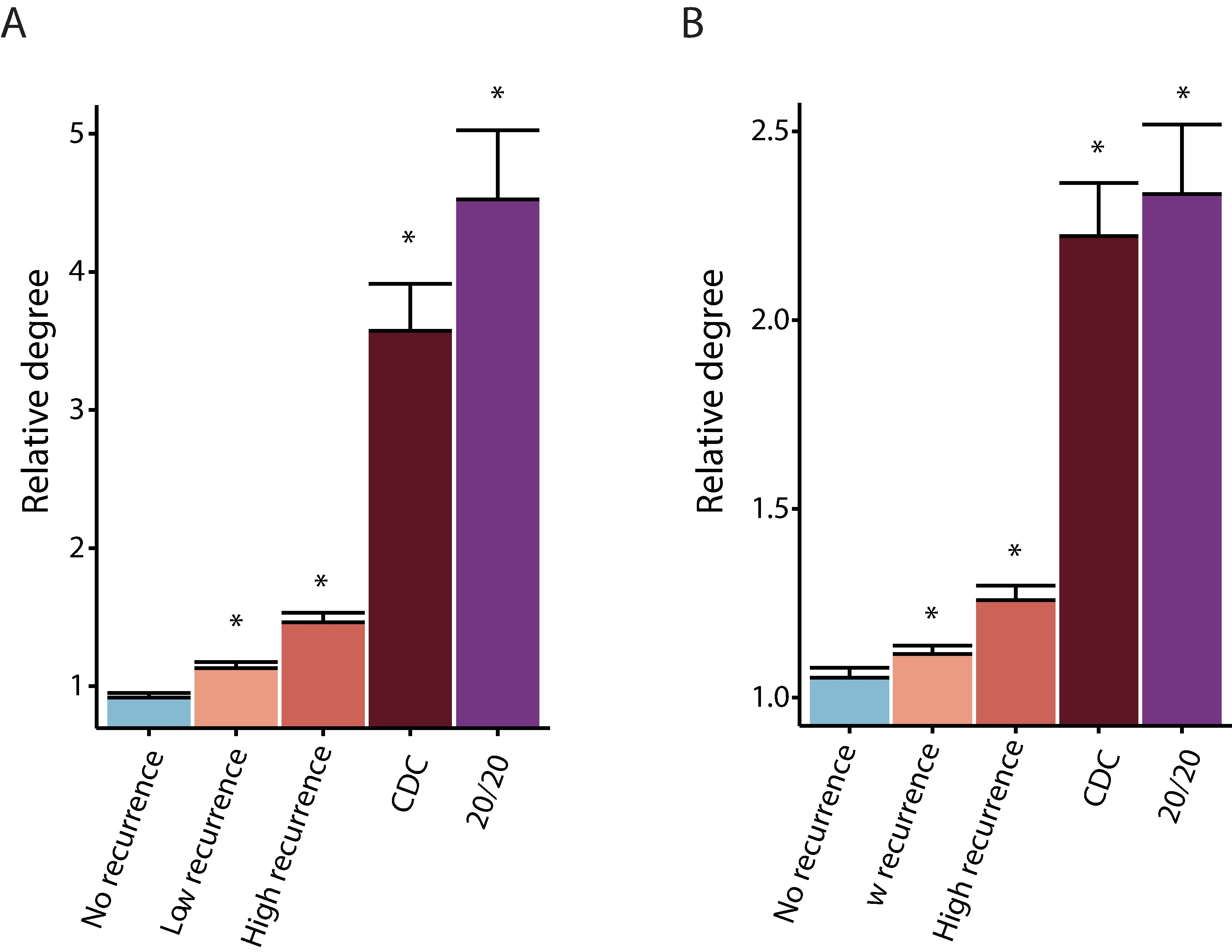

Supplement: S7 Fig — The breast and liver cancer TDs were combined. (TIF) [file pcbi.1005449.s007.tif]

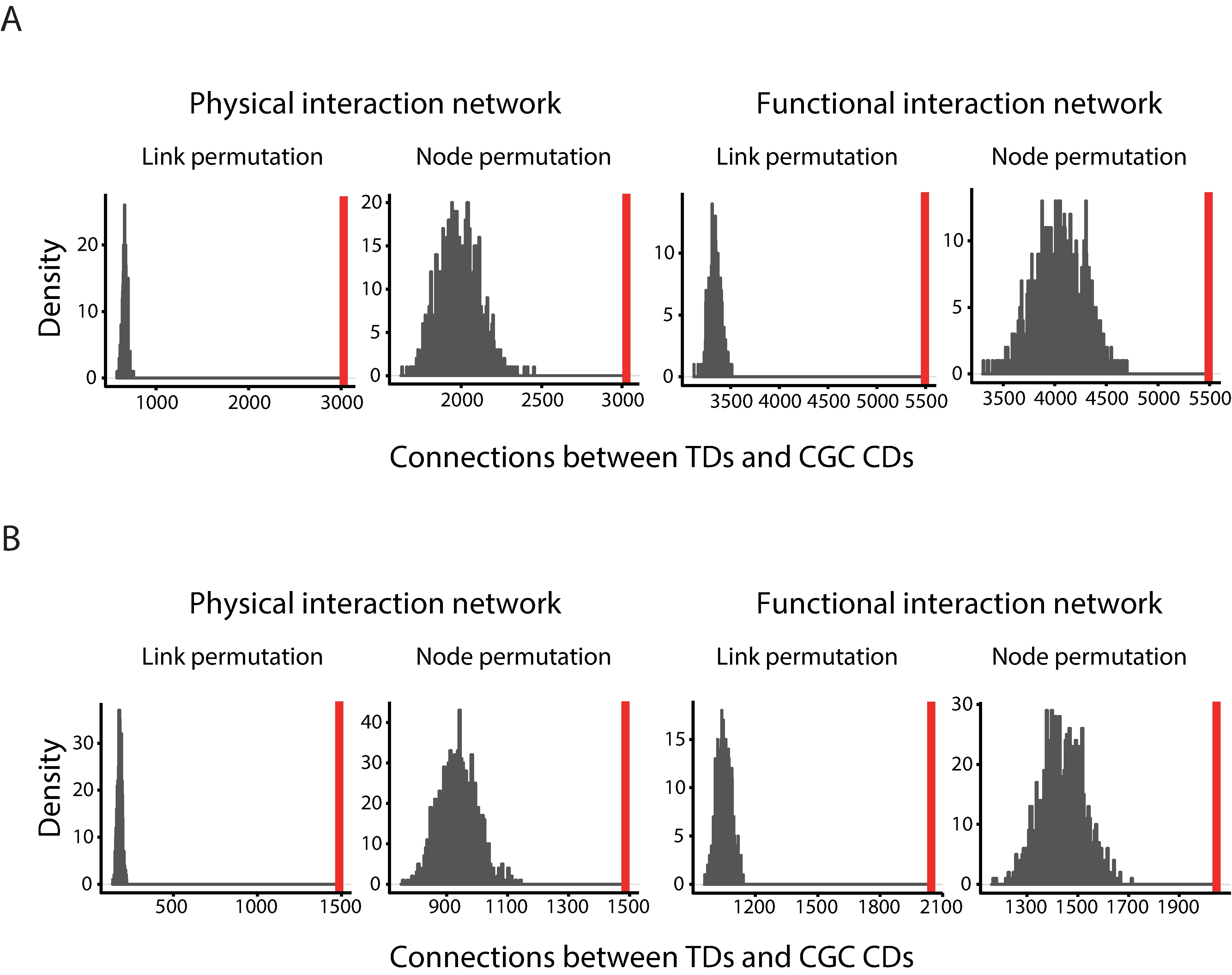

Supplement: S8 Fig — Link or node randomization was performed 1,000 times to obtain the distribution of expected number of interactions. The red lines denote the observed number of interactions. (TIF) [file pcbi.1005449.s008.tif]

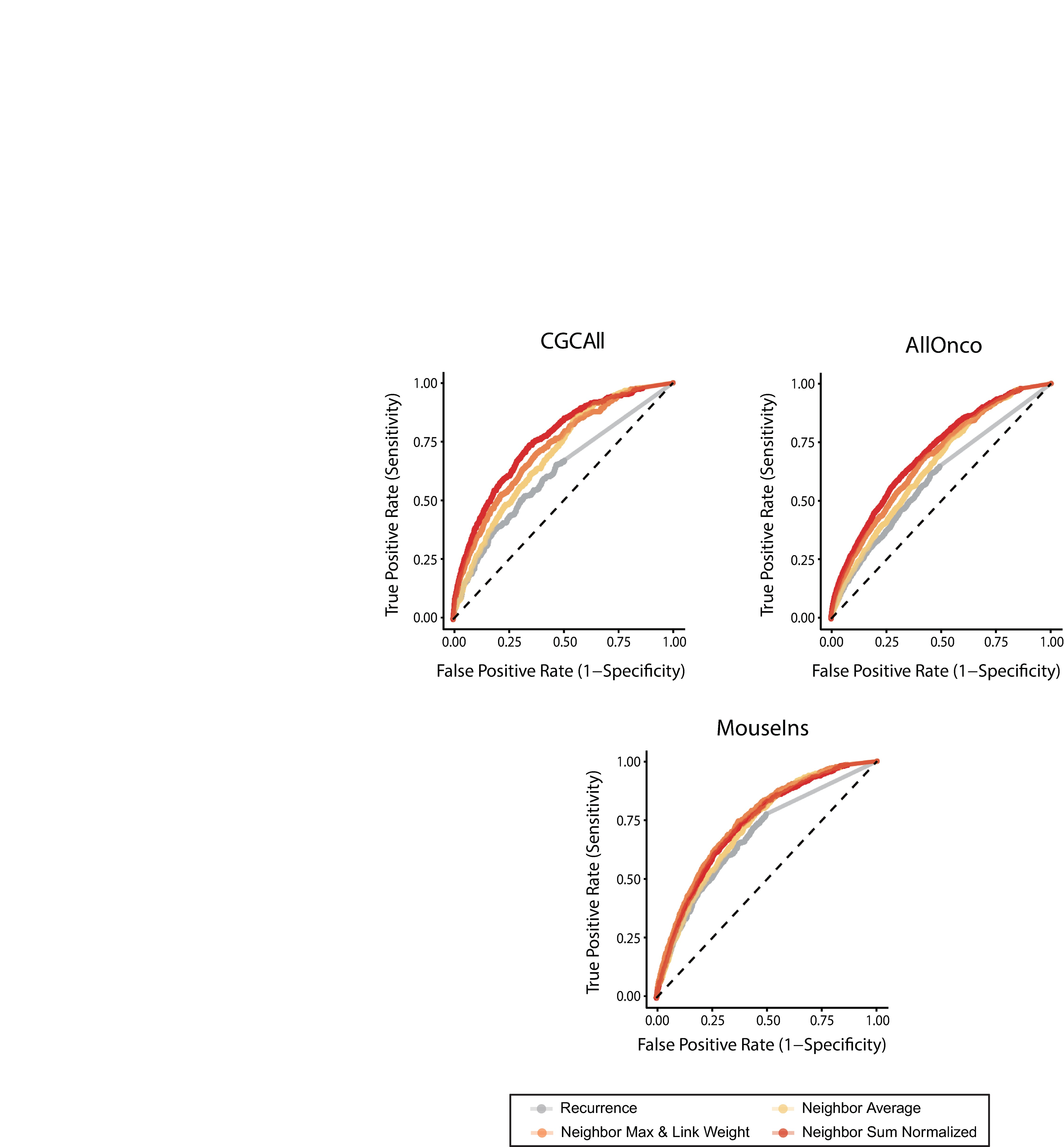

Supplement: S9 Fig — (TIF) [file pcbi.1005449.s009.tif]
